# Errors in mutagenesis and the benefit of cell-to-cell signalling in the evolution of stress-induced mutagenesis

**DOI:** 10.1098/rsos.170529

**Published:** 2017-11-01

**Authors:** Eynat Dellus-Gur, Yoav Ram, Lilach Hadany

**Affiliations:** Department of Molecular Biology and Ecology of Plants, Tel-Aviv University, Tel-Aviv 69978, Israel

**Keywords:** cell-to-cell signalling, stress-induced mutagenesis, regulation errors, populations models

## Abstract

Stress-induced mutagenesis is a widely observed phenomenon. Theoretical models have shown that stress-induced mutagenesis can be favoured by natural selection due to the beneficial mutations it generates. These models, however, assumed an error-free regulation of mutation rate in response to stress. Here, we explore the effects of errors in the regulation of mutagenesis on the evolution of stress-induced mutagenesis, and consider the role of cell-to-cell signalling. Using theoretical models, we show (i) that stress-induced mutagenesis can be disadvantageous if errors are common; and (ii) that cell-to-cell signalling can allow stress-induced mutagenesis to be favoured by selection even when error rates are high. We conclude that cell-to-cell signalling can facilitate the evolution of stress-induced mutagenesis in microbes through second-order selection.

## 1. Introduction

Stress-induced mutagenesis is the phenomenon wherein the mutation rate increases in individuals under stress. This phenomenon has been observed in numerous organisms, over the whole tree of life, from bacteria to human cells [1]. It was demonstrated both in laboratory strains [2,3] and in many other bacterial strains, including *Mycobacterium tuberculosis* [4], *Bacillus subtilis* [5,6], *Vibrio cholera* [7] and *Listeria monocytogenes* [8]. It was also observed in eukaryotes, including yeast [9,10], algae [11], nematodes [12], flies [13] and human cancer cells [14,15]. The stress-regulated mechanism for increasing mutagenesis in bacteria involves activation of error-prone DNA polymerases [16] and inhibition of DNA repair systems [6]. The increased mutagenesis is regulated by the SOS DNA-damage and other general stress responses [17], and was shown to implicate nucleoid-associated proteins (NAPs) [18] as well as sRNA [19].

Previous theoretical works showed that if one considers only deleterious mutations, then population mean fitness is not affected by stress-induced mutagenesis [20,21]. By contrast, if one also considers a low frequency of beneficial mutations, stress-induced mutagenesis increases the population mean fitness, facilitates adaptation, and is favoured by natural selection [22,23]. In these theoretical studies, all stressed individuals increase their mutation rates, while unstressed individuals do not. However, biological systems tend to be error-prone [24–26], and it can be expected that some individuals may increase their mutation rates when there is no stress, or fail to do so under stress. Therefore, errors in mutagenesis regulation might play an important role in the evolution of stress-induced mutagenesis.

Here, we study a theoretical model of stress-induced mutagenesis in the presence of errors in mutagenesis regulation. In that case, some unstressed individuals induce mutagenesis, due to regulation errors, and a similar fraction of stressed individuals fail to induce mutagenesis. First, we find that the mean fitness of a population with stress-induced mutagenesis (but not that of a population with uniform mutagenesis, where all individuals have the same mutation rate) decreases with the amount of regulation errors, suggesting that errors can limit the evolution of stress-induced mutagenesis. Next, we propose that cell-to-cell signalling might mitigate the effect of errors in mutagenesis regulation.

In bacteria, one form of cell-to-cell signalling is achieved by *quorum sensing*, a mechanism that is used to sense population density, usually to coordinate gene expression [27]. It has been suggested that intracellular errors may be corrected using population-level signalling [28]. Indeed, *quorum sensing* has been shown to regulate gene induction through population-wide signalling, and thus facilitate global coordination of gene expression in populations [29] and reduce the effects of regulation errors [30]. Recent evidence shows that *quorum sensing* is not only used to sense population density but also to regulate other functions, such as biofilm formation and virulence [31,32], contributing to stress responses [33], affecting mutation rate [34,35] and facilitating adaptation to environmental stress [36]. It has been suggested that a significant fraction of the genes regulated by *quorum sensing* are related to stress tolerance [37–39], and *quorum sensing* was demonstrated to enhance stress tolerance in several bacterial strains [40–42]. In addition, it has been shown that *quorum sensing* signals are also activated by host phosphate-limitation stress to induce virulence in *Pseudomonas aeruginosa* [33]. It is not fully clear, however, what role *quorum sensing* plays in stress response and how does it promote stress tolerance.

We thus introduce a new theoretical model of *quorum sensing* stress-induced mutagenesis (qSIM), where mutagenesis is regulated by a combination of individual stress and *quorum sensing* signal reflecting the population mean fitness: Individuals activate stress-induced mutagenesis fully when the individual stress response is triggered (accurately or erroneously) and *quorum sensing* stress signals indicate that the population is stressed. However, when either the individual or the population is not stressed, mutagenesis is not activated. Intermediate levels of population stress lead to intermediate levels of mutagenesis activation. Note that *quorum sensing* stress signal is an average over multiple stressed individuals, and is thus expected to be less noisy than the stress response of a single individual in many cases. *Quorum sensing* regulation can have two major effects. On the one hand, it can mitigate most of the effect of regulation errors on the mean fitness of stress-induced mutagenesis (SIM) populations in a constant environment. On the other hand, during adaptive evolution the mutation rate would be increased in stressed individuals, allowing rapid adaptation. Here, we use a theoretical model to explore the effect of qSIM on the evolvability and mean fitness of populations, and consider competitions between different strategies for regulation of mutagenesis: SIM, qSIM and non-mutator (NM; i.e. uniformly low rate of mutation). Our results show that the *quorum sensing* stress-induced mutagenesis strategy is favoured over stress-induced mutagenesis or non-mutator strategy over a wide parameter range. *Quorum sensing* regulation of mutation rate has not been demonstrated experimentally yet, and our results suggest that such empirical studies could be of great interest.

## 2. Model

**Figure 1.**
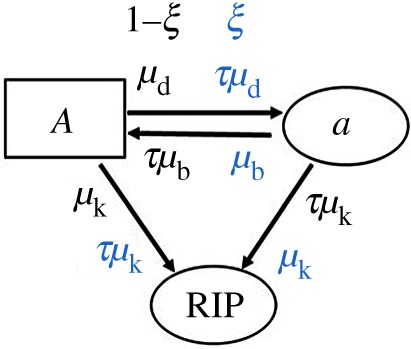
Illustration of a single locus model of stress-induced mutagenesis. In black is the fraction of population that does not exhibit errors in the regulation of mutation (1 − *ξ*), deleterious mutations during reproduction change the advantageous allele *A* to the harmful allele *a* with probability *µ*_d_; beneficial mutations change *a* to *A* with probability *τµ*_b_ due to stress-induced mutagenesis; lethal mutations, which prevent their carriers from reproducing, occur with probability *µ*_k_ in genotype *A* and with probability *τµ*_k_ in genotype *a*. In blue are the transition probabilities under regulation errors (*ξ*), where stress-induced mutagenesis increases the mutation rate of *A* carriers, but not of *a* carriers, *τ*-fold.

We model a very large asexual microbial population with non-overlapping generations. Fitness is determined by a single locus. We first model a stable environment, where individuals carrying allele *A* have fitness 1, individuals carrying the allele *a* have fitness 1 − *s* and individuals carrying a ‘lethal’ allele have fitness 0. The frequency of allele *A* after selection is denoted by *p*, and the frequency of allele *a* is 1 − *p* (as the frequency of ‘lethal’ alleles is then 0). The population mean fitness is denoted by $\;\bar{\omega }$. We model three types of mutations: (i) beneficial mutations from *a* to *A*, (ii) deleterious mutations from *A* to *a* and (iii) ‘lethal’ mutations that kill their carrier before reproduction. The rates of beneficial, deleterious and ‘lethal’ mutations are denoted by *µ*_b_, *µ*_d_ and *µ*_k_, respectively (figure 1).

The mutational strategy is determined by another locus [43], with three mutator alleles: NM (does not increase mutation rate), SIM (stress-induced mutagenesis, where mutation rates are increased by *τ* > 1 in response to individual stress) and qSIM (*quorum sensing* stress-induced mutagenesis, where mutation rates are increased by *τ* in response to the combination of individual and population stress). We examine the adaptation rate and population mean fitness of populations fixed for each of these mutator alleles, under errors in the regulation of mutagenesis in a constant environment. Then, we perform simulations of competitions between the three strategies, in the presence of errors and a changing environment.

With SIM, and in the absence of errors, we assume that all individuals carrying allele *a*, and only them, increase their mutation rate *τ*-fold. However, in the presence of errors in the regulation of mutagenesis, a fraction *ξ* of individuals carrying allele *a* do not induce mutagenesis, and similarly a fraction *ξ* of individuals carrying allele *A* do induce mutagenesis. The frequency of allele *A* in the next generation of a SIM population, *p′* is

2.1 $$\begin{array}{rl} p^{\prime } & =\frac{\left(1-\xi \right)}{\bar{\omega }}((1-\mu_{k})(1-\mu_{d})p+(1-\tau \mu_{k})(1-s)(1-p)\tau \mu_{b})\\ & \;+\frac{\xi }{\bar{\omega }}((1-\tau \mu_{k})(1-\tau \mu_{d})p+(1-\mu_{k})(1-s)(1-p)\mu_{b}). \end{array}$$

Note that *τ *= 1 represents the case of an NM population.

The population mean fitness is the weighted mean of ${\bar{\omega }}_{\mathrm{error}}$—the mean fitness of individuals exhibiting errors in mutagenesis regulation—and $\;{\bar{\omega }}_{\mathrm{correct}}$—the mean fitness of individuals with no errors in mutagenesis regulation:

2.2*a* $$\begin{array}{rl} {\bar{\omega }}_{\mathrm{correct}} & =(1-\mu_{k})p+(1-\tau \mu_{k})(1-s)(1-p), \end{array}$$

2.2*b* $$\begin{array}{rl} {\bar{\omega }}_{\mathrm{error}} & =(1-\tau \mu_{k})p+(1-\mu_{k})(1-s)(1-p) \end{array}$$

2.2*c* $$\begin{array}{rl} \;\text{and}\;\bar{\omega } & =(1-\xi ){\bar{\omega }}_{\mathrm{correct}}+\xi {\bar{\omega }}_{\mathrm{error}}. \end{array}$$

When errors in regulation are common, SIM becomes disadvantageous. Here, we introduce a new strategy—*quorum sensing* SIM (qSIM). This strategy is similar to the SIM strategy, with the only difference that the fold increase in mutation rate induced by the mutator is not a constant *τ*, but a linear function of the population mean fitness $\bar{\omega }{\;}_{\mathrm{qSIM}}$, denoted by $\tau_{\mathrm{qSIM}}$:

2.3 $$\tau_{\mathrm{qSIM}}=\frac{\left(\tau -1)({\bar{\omega }}_{\mathrm{qSIM}}-(1-\mu_{k})\right)}{\left(1-s)(1-\tau \mu_{k})-(1-\mu_{k}\right)}+1.$$

According to this function, the highest value of $\tau_{\mathrm{qSIM}}$ is obtained when all the population is unfit and population mean fitness is (1 − *s*)(1 − *µ*_k_). In that case, the mutator is fully induced and $\tau_{\mathrm{qSIM}}$ is equal to *τ*. The lowest value of $\tau_{\mathrm{qSIM}}$ is obtained when all the population is fit, population mean fitness is 1 − *µ*_k_, and $\tau_{\mathrm{qSIM}}$ is equal to 1. $\tau_{\mathrm{qSIM}}$ decreases linearly between these two extreme values of population mean fitness. Thus, when the population is far from mutation-selection balance (MSB), mutagenesis is highly induced, enabling fast adaptation. As the population is approaching MSB, mutagenesis induction is reduced proportionally, partly protecting the unstressed individuals from inducing mutagenesis due to regulation errors.

Another important aspect that remains to be explored is the effect of errors in the *quorum sensing* signalling in our model. Similar to the regulation of stress-induced mutagenesis, the *quorum sensing* signalling may also be subjected to errors. Since *quorum sensing* signals are averaged over multiple individuals, they are likely to experience lower levels of errors, but possibly not negligible. In such a case, we would expect reduction in the adaptation rate of qSIM due to individuals with lower mutagenesis rate in the adaptation stage, and reduced population mean fitness of qSIM in a constant environment due to erroneously increased mutation rate in fit individuals (when both individual stress response and population stress signal are erroneously activated).

Next, we simulate competitions between every pair of the three mutagenesis strategies: NM, SIM and qSIM. Competition simulations were performed in changing environments under varying rates of mutagenesis error. At the starting point, two populations at MSB were mixed such that each population constituted 50% of the total population. We then simulated the adaptation process of the mixed population in an environment that changes every N generations [22,23], where each environmental change flips the optimal fitness allele (from *A* to *a* or vice versa). The simulation was stopped when one of the mutator alleles reached a proportion of 90% of the mixed population, and this allele was considered the winner of the competition. If after 100 environmental changes no strategy reached 90% of the mixed population, we considered it as if no strategy won. These cases were extremely rare. We also simulated the invasion of qSIM strategy to SIM or NM populations, and the invasion of SIM to NM population under similar settings. For the full competition model, see electronic supplementary material, equations (7–10).

## 3. Results

### 3.1. Mean fitness and fixation rate

**Figure 2.**
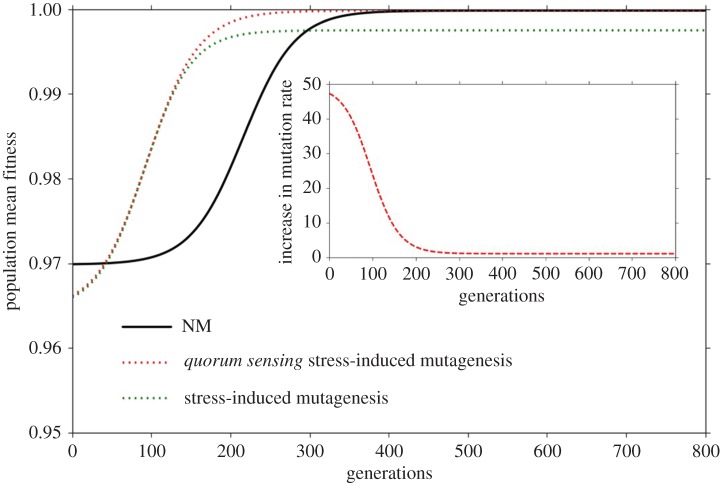
Adaptation process of the three strategies in the presence of errors. The mean fitness of populations with each strategy during an adaptation process, starting from *p*_0_ and up to MSB, with mutation rate fold increase, *τ* = 50, and mutagenesis error rate, *ξ* = 0.3. The population mean fitness of the NM strategy (black line), SIM (green dotted line) and qSIM (red dotted line). The inset shows the increase in mutation rate of the qSIM strategy (*τ*_qSIM_) during the adaptation process with *τ* = 50. All simulations use these parameter values [44–46]: selection coefficient, *s* = 0.03; lethal mutation rate, *µ*_k_= 0.00012 mutations per generation; beneficial mutation rate, *µ*_b_ = 0.00004; deleterious mutation rate, *µ*_d _= 0.00004; initial frequency of allele *A*, *p*_0_ = 0.00001.

Consistent with previous results [22], we find that when mutagenesis regulation occurs without errors (*ξ* = 0), the mean fitness of SIM populations at mutation-selection balance (MSB) increases with the mutation rate fold increase, *τ* (electronic supplementary material, figure S1). Next, we explored how errors in mutagenesis regulation affect population mean fitness and the rate of adaptation in populations with stress-induced mutagenesis. Figure 2 shows the spread of the advantageous allele *A* in populations with SIM (green line) and non-mutator (NM, i.e. *τ* = 1, black line), starting from a very low frequency of the *A* allele, up to mutation-selection balance, with frequent errors in mutagenesis regulation (*ξ* = 0.3). We found that SIM increases the adaptation rate relative to NM (as shown previously [22]), even in the presence of errors in mutagenesis regulation. On the other hand, with frequent errors, the mean fitness of SIM at MSB is lower than that of NM. Altogether, although stress-induced mutagenesis increases the adaptation rate, it carries a cost in terms of the population mean fitness at mutation-selection balance when errors are frequent.

These results suggest that although the SIM strategy shows robustness to errors, it might be less advantageous when the regulation of mutagenesis is subject to some error levels. We next present results for *quorum sensing* SIM (qSIM), wherein the mutagenesis induction level is a function of *quorum sensing* signals, indicating the stress level of the population (equation (2.3)). Figure 2 (dotted red line) shows the adaptation process in populations with qSIM under errors in mutagenesis regulation. Unlike SIM populations, the mean fitness of qSIM populations at MSB is barely affected by errors in mutagenesis regulation, and is very similar to that of NM (black line). Moreover, the adaptation rate of populations with qSIM is very similar to that of populations with SIM.

**Figure 3.**
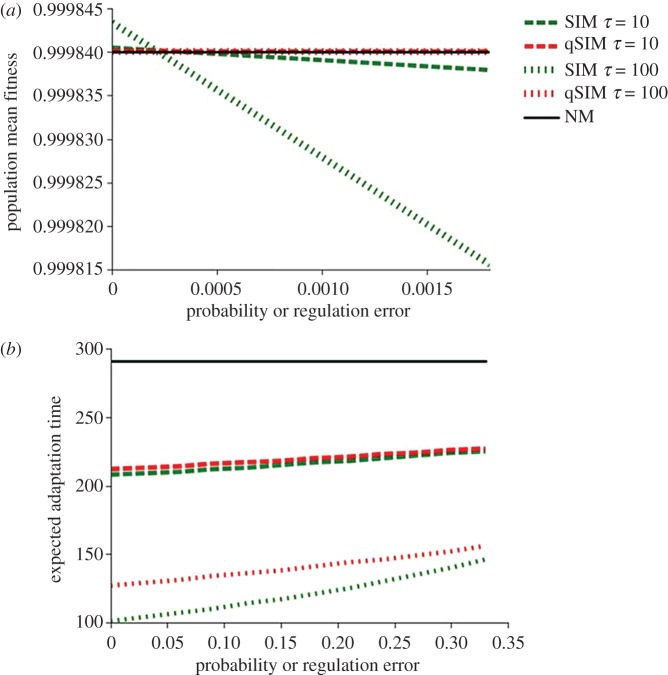
The effect of regulation errors and mutational strategies on mean fitness and adaptation time. (*a*) The mean fitness at MSB with varying rates of regulation error of populations with NM (black line), SIM (*τ* = 10, green dashed line; *τ* = 100, green dotted line), and qSIM (*τ* = 10, red dashed line; *τ* = 100, red dotted line). (*b*) Adaptation time—the number of generations it takes for the frequency of allele *A* to go from 0.00001 to 0.9 with varying rates of mutagenesis error. A smaller number of generations indicates faster adaptation rate. All simulations use these parameter values [44–46]: selection coefficient, *s* = 0.03; lethal mutation rate, *µ*_k_ = 0.00012 mutations per generation; beneficial mutation rate, *µ*_b_ = 0.00004; deleterious mutation rate, *µ*_d _= 0.00004; initial frequency of allele *A*, *p*_0_ = 0.00001.

We further analyse the mean fitness at MSB for a range of error rates. As errors in nature can be the outcome of noise in many different systems, from mRNA level to protein transcription [24] or DNA binding [26], we test a large range of error rates. Figure 3*a* shows the mean fitness of populations with SIM, qSIM and NM strategies (green, red and black lines, respectively) at MSB under a range of error rates and different mutagenesis induction levels. The mean fitness of populations with SIM decreases with increasing error rates, and the decrease in mean fitness at MSB is more severe when *τ* increases. The mean fitness of qSIM at MSB, under the same conditions, is very similar to that of NM and, as expected, is barely affected by errors.

In electronic supplementary material, figure S1*a* we explore the relative mean fitness of populations with SIM, given by $(\bar{\omega }{\;}_{\mathrm{SIM}}-\;\bar{\omega }{\;}_{\mathrm{NM}})/\;\bar{\omega }{\;}_{\mathrm{NM}}$, under varying error rates and mutagenesis induction levels. We further find, through numerical analysis, the critical error rate, *ξ*_crit_, for which the mean fitness of SIM populations at MSB is equal to that of the NM populations at the MSB. Above that critical rate of error in mutagenesis regulation, *ξ*_crit_, the mean fitness of populations with NM is higher than that of populations with SIM, but the difference is small.

We then calculated the relative mean fitness of populations with qSIM, and found the critical error rate for which it is still beneficial to induce mutagenesis, *ξ*_crit_ (electronic supplementary material, figure S1*b*). Under the parameters studied [22] *ξ*_crit_ for qSIM and for SIM was similar. However, the reduction in mean fitness due to qSIM is much smaller than due to SIM: the relative difference in mean fitness between populations with SIM and ones with NM is in the range of 10^−6^, while for populations with qSIM, the relative difference from NM is in the range of 10^−9^.

Next, we explore the effect of different mutational strategies on the expected adaptation time, measured as the number of generations it takes for the advantageous allele *A* starting at 0.001% to reach 90% of the population. Figure 3*b* shows the fixation time in populations with NM, SIM and qSIM. Although the fixation time is a bit longer in populations with qSIM than in populations with SIM (because mutagenesis is induced to a slightly lower level with qSIM, unless everybody is stressed), both strategies exhibit dramatically faster adaptation than populations with NM, and the difference further increases with the mutation rate fold increase, *τ*. The effect of errors in the regulation of mutagenesis on the rate of adaptation is negligible.

### 3.2. Competitions between mutational strategies

Overall, when no errors in mutagenesis regulation occur, we see that populations with SIM exhibit the highest fixation rate and the highest mean fitness at MSB. However, this changes in the presence of frequent errors: in terms of mean fitness at MSB, populations with NM dominate, populations with qSIM fall slightly behind, whereas populations with SIM have significantly lower mean fitness (due to increased mutation rate in well-adapted individuals as a result of errors). In terms of adaptation rate, populations with SIM adapt the fastest, populations with qSIM lag slightly behind, while the rate of fixation in populations with NM is much slower than both, regardless of the presence of errors in mutagenesis regulation. Therefore, it is hard to estimate which strategy would be favoured in direct competition.

**Figure 4.**
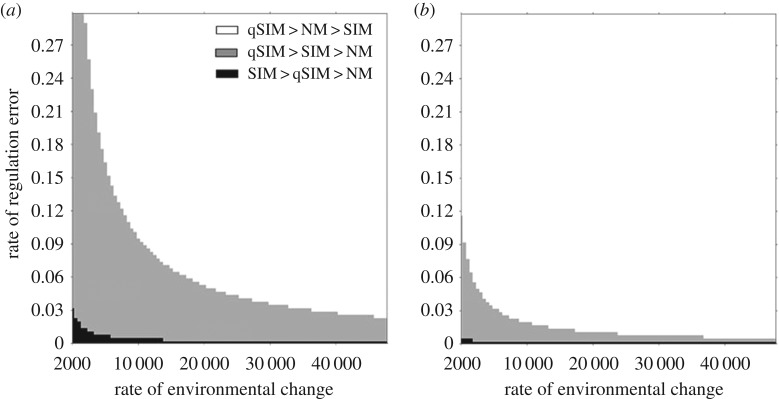
Simulated competitions between the three strategies. Simulations of pairwise competitions between the mutagenesis strategies under varying rates of mutagenesis error and environmental changes. qSIM is favoured over a wide parameter range (white and grey); otherwise, SIM is favoured when environmental changes are rare and the rate of regulation errors is low (black). (*a*) Mutation rate fold increase, *τ* = 10, (*b*) *τ* = 100. All simulations use these parameter values [44–46]: selection coefficient, *s* = 0.03; lethal mutation rate, *µ*_k_ = 0.00012 mutations per generation; beneficial mutation rate, *µ*_b_ = 0.00004; deleterious mutation rate, *µ*_d _= 0.00004; initial frequency of allele *A*, *p*_0_ = 0.00001.

To answer this question, we simulated pairwise competitions between the different strategies in slowly changing environments (electronic supplementary material, equations (4–7)). We ran the competitions under varying error rates, and under different rates of environmental change. We considered three types of competitions: SIM-NM, qSIM-NM and qSIM-SIM. Figure 4 summarizes the outcomes of all competitions, under two levels of mutagenesis induction. First, qSIM wins in competitions with NM over the entire range of conditions tested. Second, qSIM wins over all strategies in all the grey and white areas. When considering competitions between SIM and qSIM, we see that when the error rate is low enough, SIM is the favoured strategy (black area), and the critical value of error increases with the rate of environmental change, due to the higher adaptation rate of the SIM strategy. However, for higher error rates or slower rates of environmental change, the qSIM strategy wins. For intermediate error values, qSIM is favoured over SIM and SIM over NM (grey area), and for even higher error values, qSIM is favoured over NM, and NM over SIM (white area). Similar results were observed in competitions where one strategy (the ‘invader’) was initially rare. We tested qSIM strategies invading populations of NM or SIM, and an SIM strategy invading a population of NM, and the results were qualitatively similar to the results obtained starting at 50%, where the rest of the parameters are kept equal (electronic supplementary material, figure S2). Note that if the rate of environmental changes is so slow that the population spends most of the time at MSB (much beyond the range presented here, down to one environmental change every 50 000 generations) NM would eventually win. However, we find that even rare changes in the environment can be enough to render NM less favourable.

## 4. Discussion

We studied the effects of errors in mutagenesis regulation on the evolutionary advantage of stress-induced mutagenesis (SIM), building upon previous studies [22]. Using a theoretical model, we showed that when errors in mutagenesis regulation are frequent enough, SIM is less advantageous in a constant environment. We thus suggest a new strategy, *quorum sensing* stress-induced mutagenesis (qSIM), which combines individual and population stress signals to regulate mutagenesis using *quorum sensing* cell-to-cell signalling. Indeed, populations with qSIM were less affected by errors in mutagenesis regulation. Altogether, in the presence of both frequent errors in mutagenesis regulation and rare environmental changes, qSIM is favoured over both SIM and non-mutators (NM). However, we are well aware that the conditions tested in our simulations are only an approximation of the conditions in nature [44]. For example, the rate of environmental changes and the selection pressure, as well as other parameters of the model. In particular, a critical parameter in our model is the error rate in the regulation of mutagenesis. If this error rate evolved to be very low, we would expect SIM, and not qSIM, to be common in the world. Empirical estimates of that parameter in different species would be very interesting for our work.

Previous theoretical studies have shown that introducing errors into the regulation of stress responses can result in reduced population mean fitness for a population employing stress-induced variation. One example is fitness-associated sex (FAS) [47], wherein sexual reproduction is correlated with stress. It has been shown that when the information about the stress condition of an individual is inaccurate, the population mean fitness decreases. Another study [23] has shown that mixed mutagenesis strategies, that are partly constitutive and partly stress-induced, cause a reduction in population mean fitness relative to the standard SIM. These mixed strategies can represent cases where the information about the individual's condition is correct, but mutagenesis induction is leaky, i.e. induced regardless of individual's condition.

Our model can be extended in several ways. A multi-locus model, which involves a large set of fitness affecting genes and different selection coefficients, might provide a more complete picture of the effects of regulation errors and cell-to-cell signalling on the evolution of stress-induced mutagenesis. In addition, cell-to-cell signalling might have a cost, which is not taken into account in our model [48]. This can potentially increase the benefits of strategies that do not invest energy in signalling, such as SIM and NM, over the qSIM strategy. Yet, cell-to-cell signalling in bacteria has evolved due to other reasons [33], and qSIM is probably using information that is already available in the cell.

Interestingly, we find that although the qSIM strategy does not exhibit the fastest adaptation rate, nor the highest mean fitness in mutation-selection balance, it might be the strategy most often favoured by natural selection, specifically when regulation errors are frequent. Therefore, qSIM can help explain the wide occurrence of stress-induced mutagenesis in microbial populations [1–3], even in the face of errors in mutagenesis induction. It would thus be interesting to explore qSIM empirically, as no clear evidence of such mechanism has been observed in nature yet. qSIM could be tested experimentally by exploring the mutation rate of bacteria strains exhibiting stress-induced mutagenesis, under different stress levels and in the presence of gradual levels of *quorum sensing* signals. It will also be interesting to explore whether *quorum sensing* signals activate genes in the known stress-regulated mechanisms for increasing mutagenesis in bacteria [6,16–19,49].

## Supplementary Material

Figure S1 and Model extantion

## Supplementary Material

Model Code

## Data accessibility

The Python code used to produce the figures is deposited as electronic supplementary material.
