# Supplementary material for: Errors in mutagenesis and the benefit of cell-to-cell signalling in the evolution of stress-induced mutagenesis
Source: R Soc Open Sci. 2017 Nov 1;4(11):170529. doi: 10.1098/rsos.170529 (PMC5717628; doi:10.1098/rsos.170529)

## Supplementary


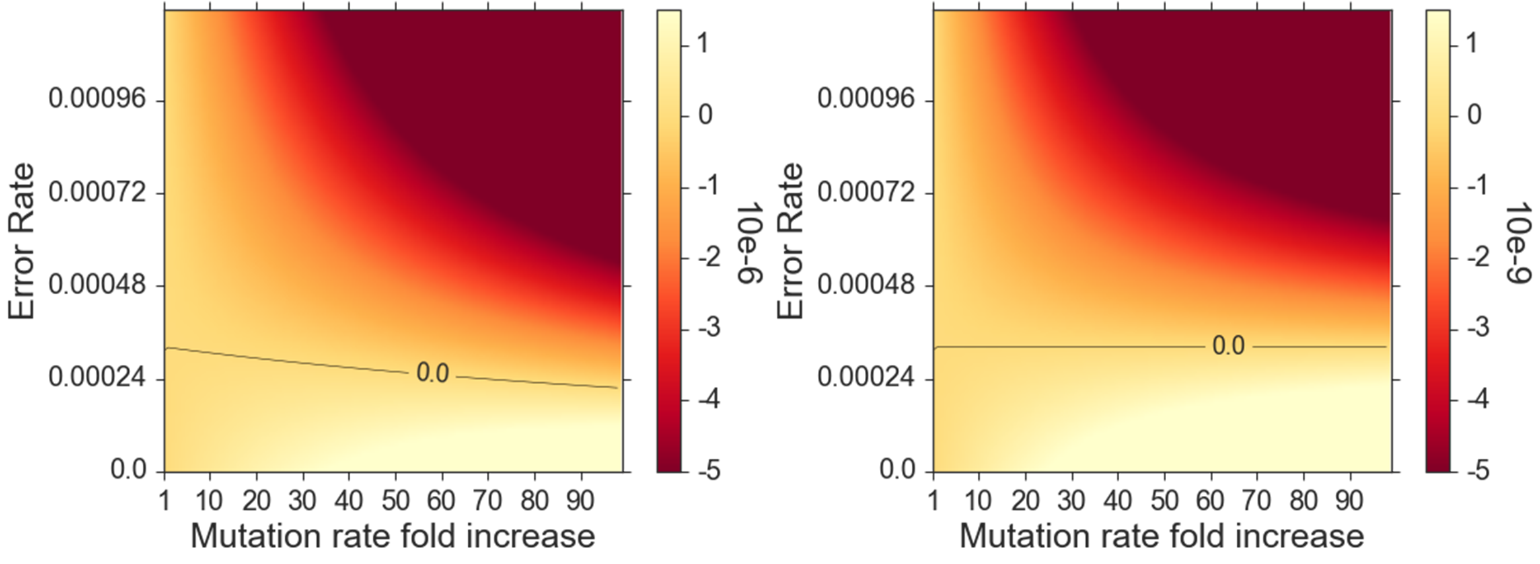


B

A

**Figure S1. The mean fitness difference between populations with SIM or qSIM relative to populations with NM.** The relative difference in population mean fitness is $\frac{\bar{\omega}_{x}}{\bar{\omega}_{NM}}-1$ where *x* is SIM or qSIM. ξ_crit_ is the regulation error rate at the zero contour line, where the mean fitness of population with NM is equal to that of populations with SIM (A) or qSIM (B). Note that the scale in of (B) is 3 orders of magnitude smaller than that of (A). All simulations use these parameter values [44-46]: selection coefficient, s=0.03; lethal mutation rate, µ_k_= 0.00012 mutations per generation; beneficial mutation rate, µ_b_= 0.00004; deleterious mutation rate, µ_d_ = 0.00004; initial frequency of allele *A*, *p_0_* = 0.00001.


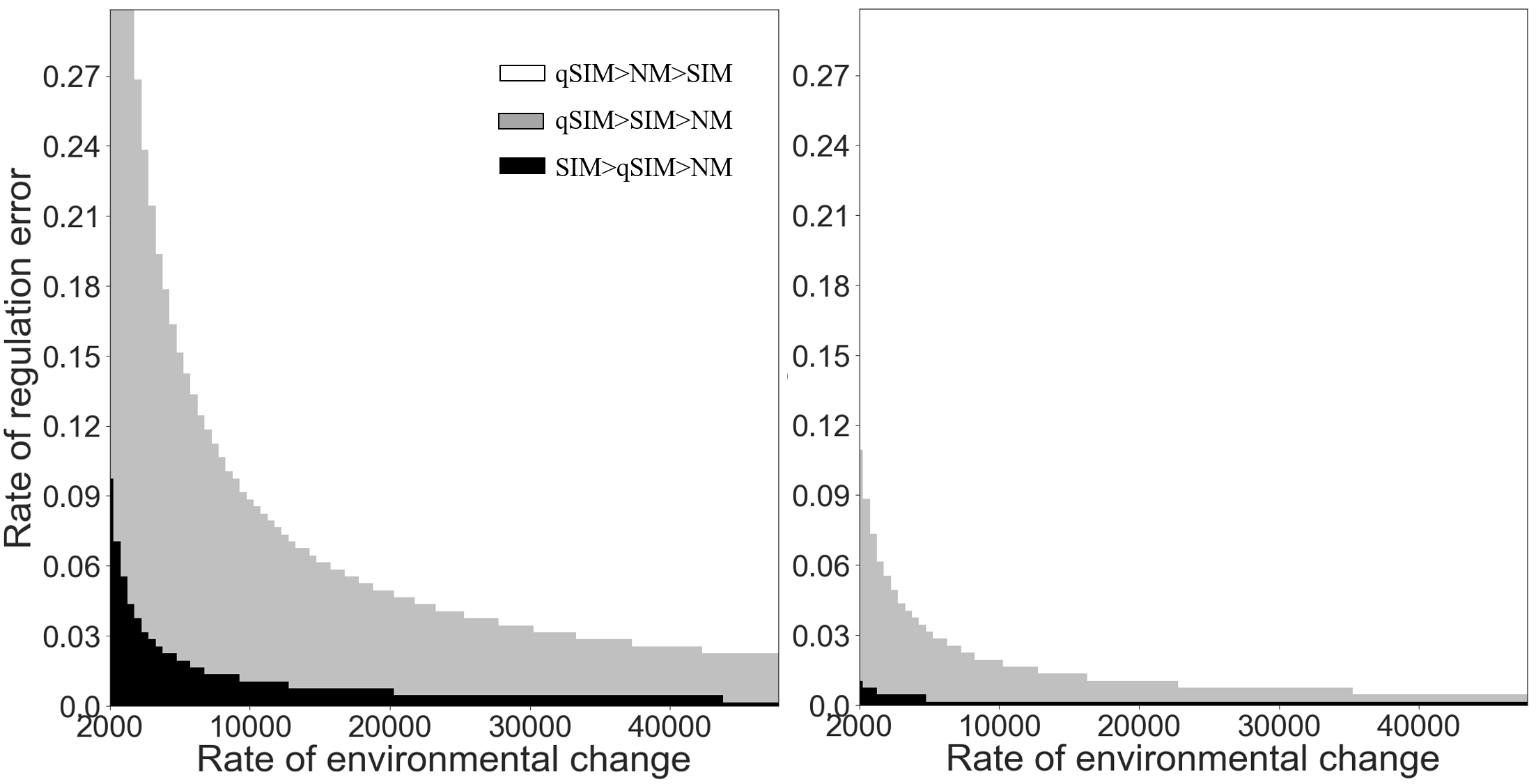
**Figure S2. Simulated competitions and invasion between the three strategies.** Simulations of invasion of the different mutagenesis strategies under varying rates of mutagenesis error and environmental changes. At the starting point, two populations at MSB were mixed such that the invading population constituted only 1% of the total population (simulation includes invasion of qSIM to NM or SIM, and of SIM to NM). The simulation was stopped when one of the mutator alleles reached a proportion of 99.99% of the mixed population, and this allele was considered the winner of the competition. Otherwise the simulations were performed as described in the competition simulations. qSIM is favored over a wide parameter range (white and gray); otherwise SIM is favored when environmental changes are rare and the rate of regulation errors is low (black). **(A)** Mutation rate fold increase, τ = 10, **(B)** τ = 100. All simulations use these parameter values: selection coefficient, s=0.03; lethal mutation rate, µ_k_= 0.00012 mutations per generation; beneficial mutation rate, µ_b_= 0.00004; deleterious mutation rate, µ_d_ = 0.00004; initial frequency of allele *A*, *p_0_* = 0.00001.

B

A

***Full model for competition between mutagenesis strategies***

The allele frequencies of the two competing strategies, for example strategies *x* and *y*, are denoted by *p*_x_ for allele *A* and *q_x_* for allele *a* in strategy *x*, *p*_y_ for allele *A* and *q_y_* for allele *a* in strategy *y,* so that:


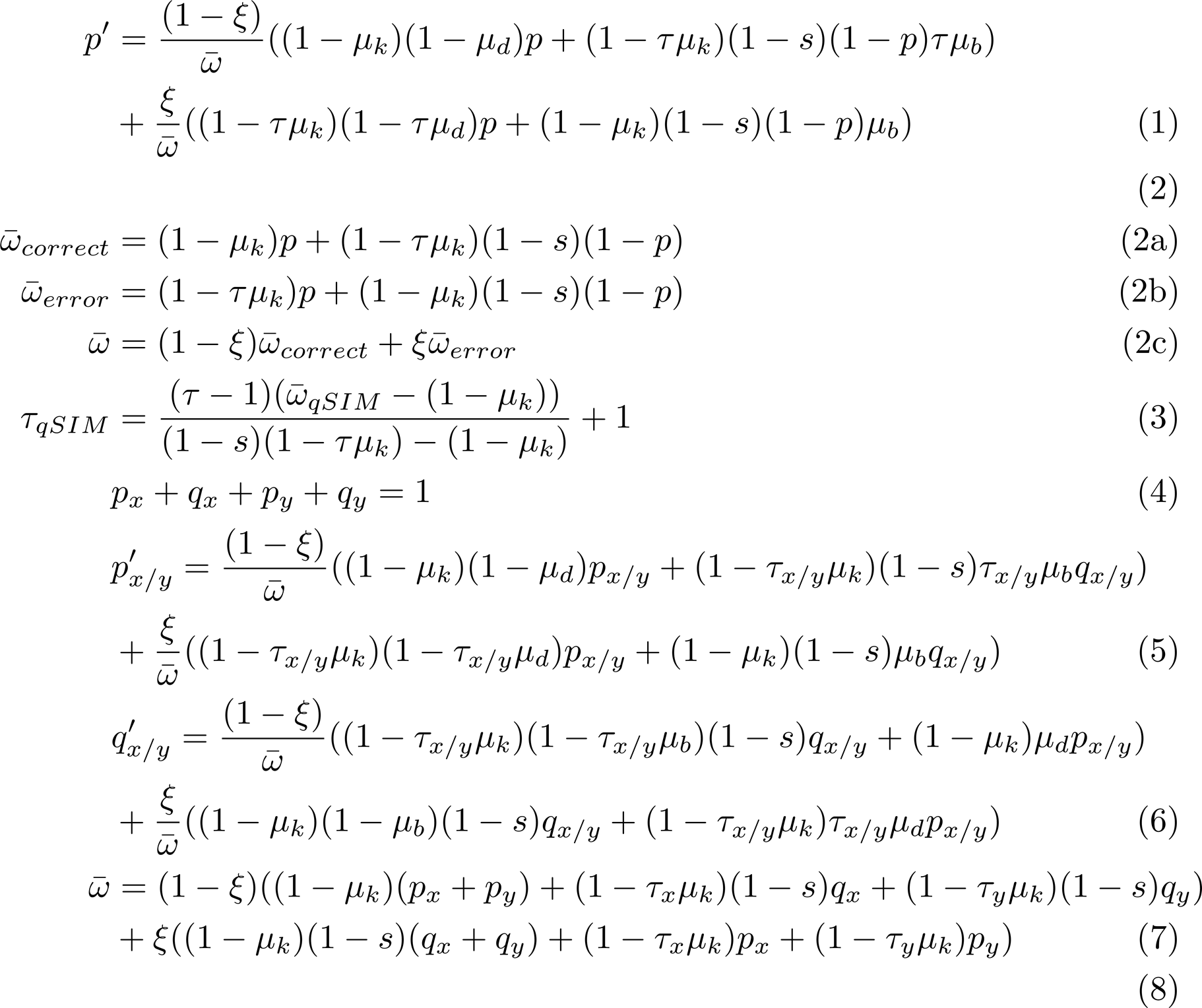


The allele frequency of *A* allele in one of the strategy, *x* (and similarly for strategy *y*), after selection is as in equation 1, but with *q_x_* replacing *1-p*:


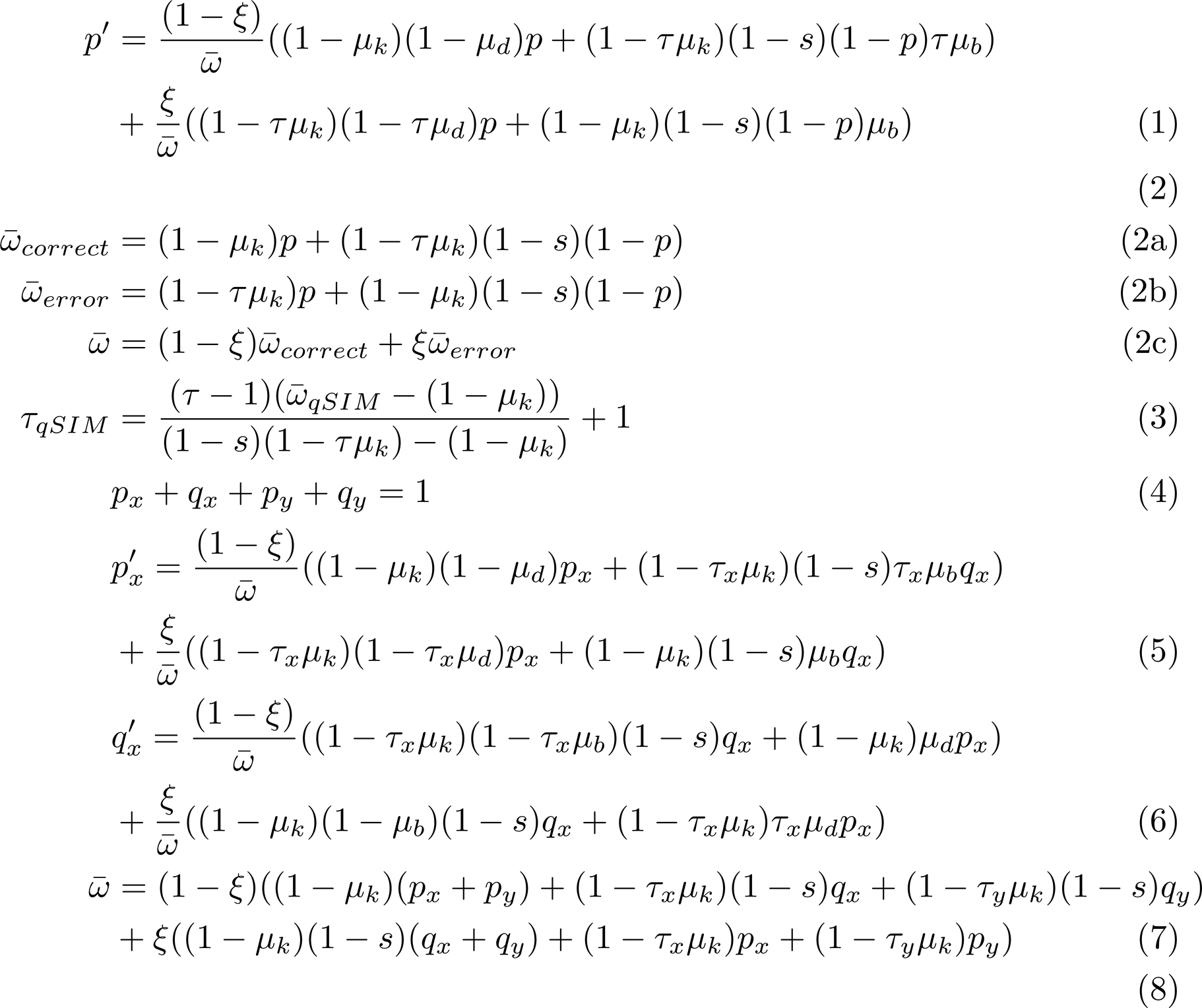


Similarly, frequency of *a* allele in strategy *x* (or *y*) after selection is denoted by:


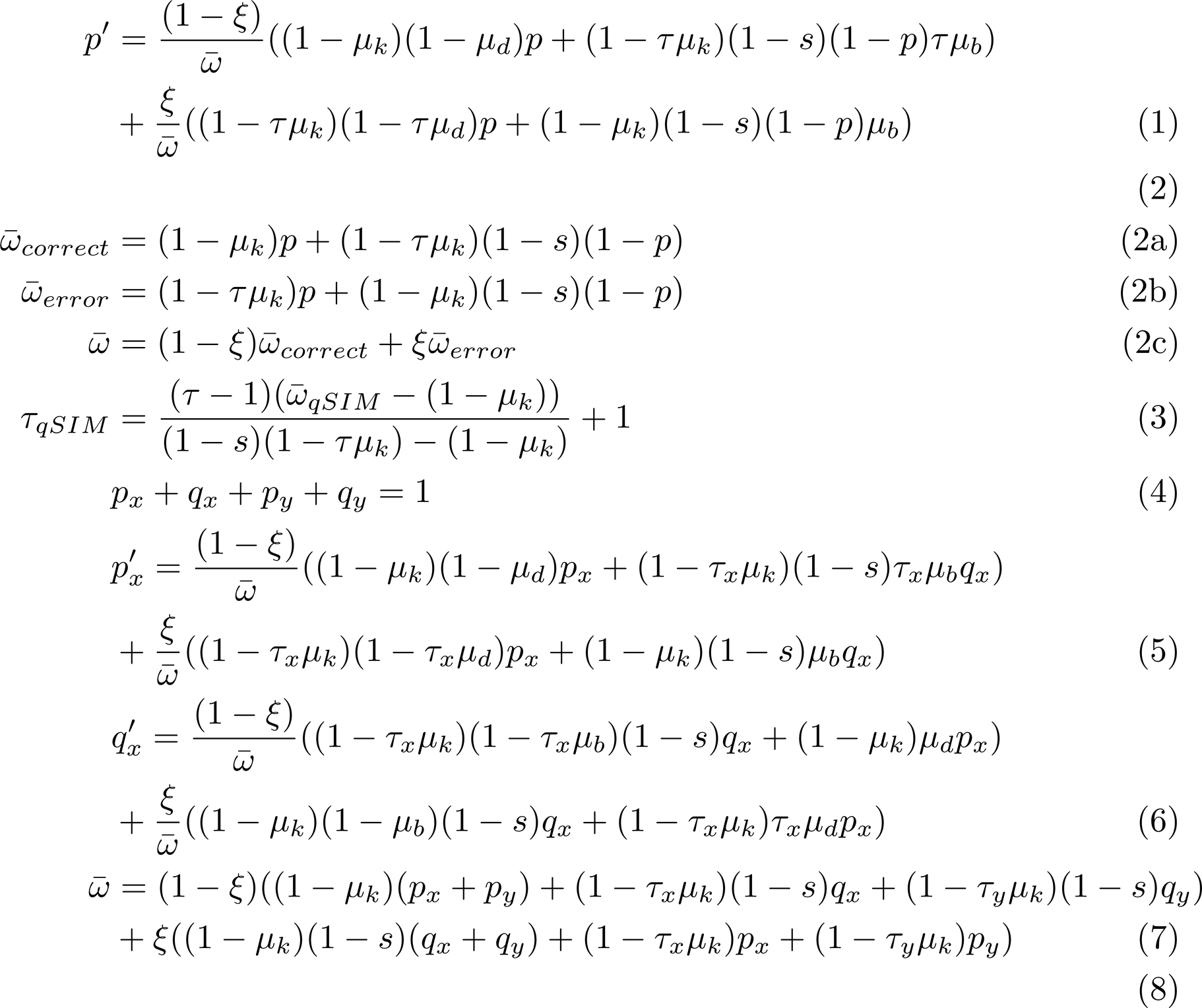


And the population mean fitness is as in equations 2, but with allele frequencies of both strategies:


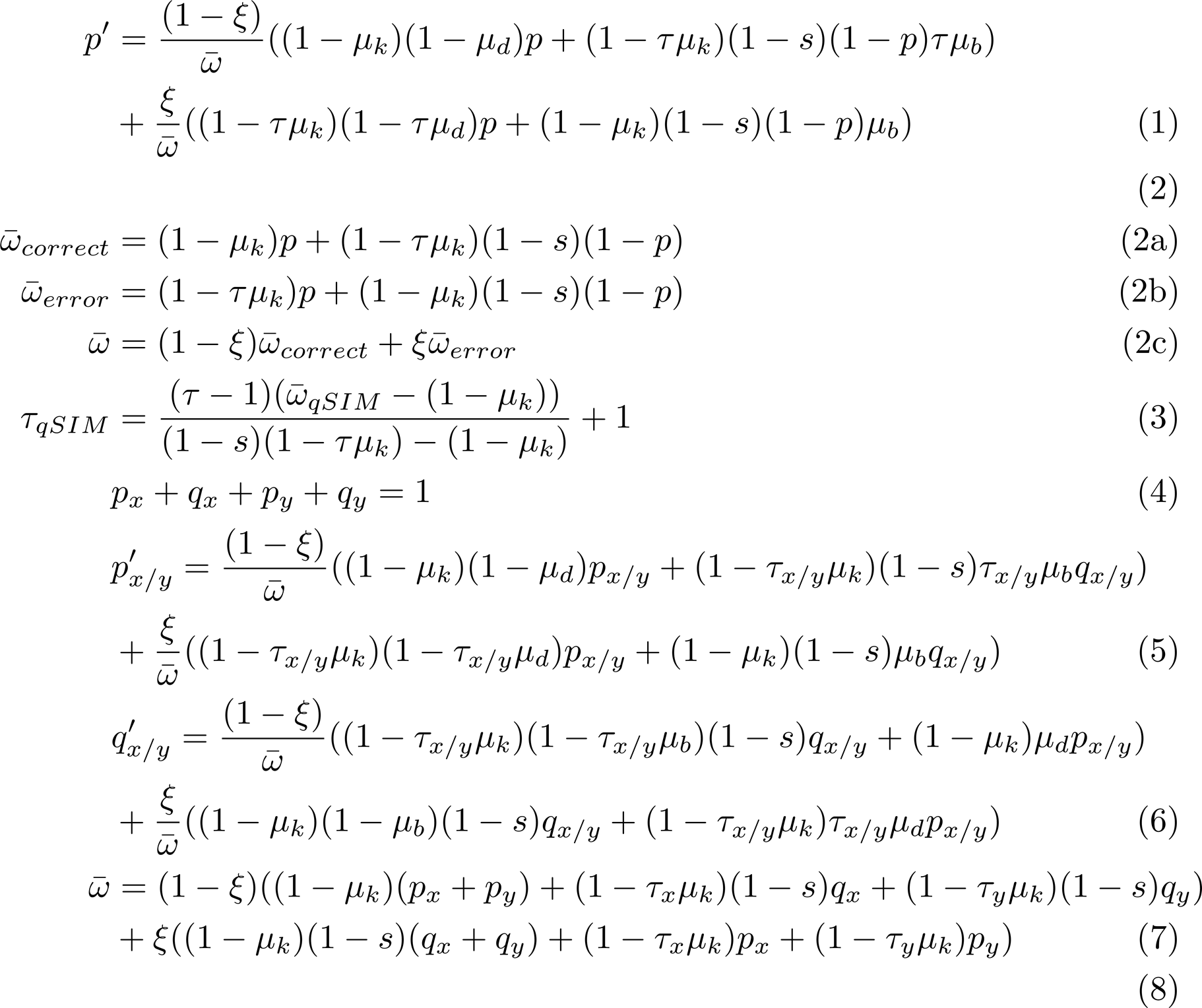

Supplement: Figure S1 and Model extantion [file rsos170529supp1.docx]
